# The Ability of Patients With Schizophrenia to Comprehend and Produce Sincere, Deceitful, and Ironic Communicative Intentions: The Role of Theory of Mind and Executive Functions

**DOI:** 10.3389/fpsyg.2019.00827

**Published:** 2019-05-08

**Authors:** Francesca M. Bosco, Laura Berardinelli, Alberto Parola

**Affiliations:** ^1^Department of Psychology, University of Turin, Turin, Italy; ^2^Institute of Neuroscience, Turin, Italy; ^3^Department of Mental Health, A.S.L. “Città di Torino”, Turin, Italy

**Keywords:** schizophrenic pathology, pragmatics, theory of mind, executive function, schizophrenia

## Abstract

Patients with schizophrenia are often described as impaired in several cognitive domains. Specifically, patients with schizophrenia often exhibit problems in solving tasks requiring theory of mind (ToM), i.e., the ability to ascribe mental states to oneself and others, communicative-pragmatic ability, i.e., the ability to use language and non-verbal expressive means to convey meaning in a given context, and executive functions (EF). This study aims to investigate the role of cognitive functions, such as general intelligence, selective attention, processing speed, and especially EF (working memory, cognitive flexibility, inhibition, and planning), and ToM in explaining the performance of individual with schizophrenia in comprehending and producing communicative acts expressed with different communicative intentions (i.e., sincere, deceitful, and ironic), and realized through linguistic and extralinguistic/non-verbal expressive means. Thirty-two patients with schizophrenia and an equal number of healthy controls performed tasks aiming to investigate their capacity to comprehend and produce sincere, deceitful, and ironic communicative acts in addition to a series of cognitive tasks evaluating EF and ToM. The results indicated that individuals with schizophrenia performed worse than the controls in the comprehension and production of all pragmatic phenomena investigated, as well as in all the cognitive functions examined. The patients with schizophrenia also exhibited an increasing trend of difficulty in comprehending and producing sincere, deceitful, and ironic communicative acts expressed through either linguistic or extralinguistic means. Furthermore, a multiple regression analysis of the patients’ performance on the pragmatic tasks revealed that overall, the role of attention, general intelligence, and processing speed did not appear to significantly explain the patients’ communicative-pragmatic performance. The inclusion of EF into the analysis did not contribute to increase the explained variance of the patients’ ability to comprehend and produce the various pragmatic phenomena investigated. Only the addition of ToM could significantly increase the explained variance, but only in the comprehension and production of deceit expressed by language and the production of sincere communicative acts, also limited to linguistic production. We conclude that neither EF nor ToM are able to explain the decreasing trend detected in the patients’ pragmatic performance.

## Introduction

Since Frith’s (1992) theoretical proposal, numerous studies in the literature have reported a theory of mind (ToM) impairment in patients with schizophrenia (Abu-Akel, 1999; Sarfati and Hardy-Baylé, 1999; Bosco et al., 2009; Bliksted et al., 2014; Csukly et al., 2014; Bechi et al., 2018; Vaskinn et al., 2018), which is a difficulty attributing mental states, such as intentions, desires and beliefs, to themselves and others (Premack and Woodruff, 1978). Several reviews and meta-analyses have confirmed this finding. For example, Harrington et al. (2005) conducted a review of studies comparing persons with schizophrenia and healthy controls performing ToM tasks, and the authors pointed out that most previous researchers showed that individuals with schizophrenia performed significantly worse than the controls in at least one of the tasks investigated. Additionally, a previous meta-analysis of Sprong et al. (2007) showed that the performance of patients with schizophrenia significantly differed from the average performance of healthy controls; furthermore, demographic variables, such as age, educational level and gender, did not affect such results. Bora et al. (2009) conducted a more controlled meta-analysis and reached a similar conclusion. Brune (2005) reviewed previous studies investigating ToM deficits in individuals with schizophrenia while considering the role of general intelligence, i.e., IQ, and concluded that the ToM deficit was not a consequence of general cognitive impairment. Further studies have reported that after controlling for other cognitive functions, such as general intelligence, attention and memory, the differences in ToM tasks between individuals with schizophrenia and healthy controls remained significant (see also Brüne and Bodenstein, 2005; Mo et al., 2008; Bliksted et al., 2014).

However, other authors have argued that a deficit in executive functions (EF) should be considered the core cognitive impairment of the disease and that such deficits are primary (Weickert et al., 2000; Reichenberg and Harvey, 2007). EF refer to abilities, such as *shifting* to switch attention among multiple tasks; *updating* to manipulate information in working memory (WM); and *inhibition* to suppress pre-potent responses (Miyake et al., 2000). This set of complex abilities is associated with brain activity in the frontal lobe (Eisenberg and Berman, 2010). These abilities allow a person to plan behavior flexibly and effectively, make decisions in a sequential and hierarchical order, and engage in goal-directed behavior flexibly, adapting behavior to the specific context. Numerous studies have shown that all EF mentioned above are severely impaired in schizophrenia (for a review, see Reichenberg and Harvey, 2007: Orellana and Slachevsky, 2013). Previous studies have evaluated the role of EF in explaining ToM impairment in patients with schizophrenia (for a review, see Bosco and Parola, 2017). Pickup (2008) analyzed studies investigating both ToM and EF deficits in individuals with schizophrenia; the author found that patients with schizophrenia have deficits in mentalizing and other cognitive abilities and revealed a strong correlation between these abilities. However, the author also found that most studies, even after controlling for EF, confirmed the residual presence of ToM deficits. Pickup (2008) concluded that in patients with schizophrenia, ToM deficits do not depend on executive functioning.

Impairment in communicative-pragmatic ability is another well-documented impairment associated with schizophrenia (Cummings, 2017; Bucca, 2018). Several studies have pointed out that the performance of persons with schizophrenia significantly differed from that of controls in the comprehension of communicative acts that implied a gap between the literal and communicative meaning, such as indirect speech acts (Corcoran, 2003), irony (Langdon et al., 2002; Tavano et al., 2008; Varga et al., 2018) and other forms of figurative language, such as metaphors, idioms and proverbs (Schettino et al., 2010; Haas et al., 2014; Moro et al., 2015). Patients with schizophrenia also show difficulty in other pragmatic domains, such as recognition and recovery of communicative failures (Bosco et al., 2012), narrative production (Marini et al., 2008), deceit (Frith and Corcoran, 1996), and scalar implicatures (Wampers et al., 2018).

Typically, the studies in the literature have focused on the ability of patients with schizophrenia to use language (e.g., Linscott, 2005; Moro et al., 2015; Thanh et al., 2017; Corcoran et al., 2018; Pauselli et al., 2018), while non-verbal, extralinguistic and paralinguistic modalities have been generally overlooked (see Cohen et al., 2014; Parola et al., 2018, 2019b). However, Colle et al. (2013) assessed the ability of individuals with schizophrenia to comprehend and produce different types of pragmatic phenomena, such as direct and indirect communicative acts, deceit and irony, and investigated extralinguistic and paralinguistic modalities in addition to language. The results of the study pointed out that the participants with schizophrenia performed significantly worse than the healthy controls in all investigated tasks expressed in both expressive modalities. By comparing some of the pragmatic phenomena investigated, the authors detected a pattern of increasing difficulty in the comprehension and production of direct and indirect sincere communicative acts, which were the easiest tasks to perform, followed by the comprehension and production of deceit and irony, which were the most difficult tasks to solve. The authors explained these results in terms of the demands on the increasing inferential ability involved in the various types of tasks. Inferences refer to the ability to bridge the gap between what a speaker literally said and what s/he wished to communicate, as in the case of indirect speech acts, irony and other forms of figurative language (see, for example, Grice, 1975; Searle, 1979). Inferential abilities play a crucial role in the pragmatic domain, i.e., the use of language and other expressive means to convey a communicative meaning (Levinson, 1983).

Colle et al. (2013) explained the increasing trend of difficulty in the comprehension and production of sincere, ironic and deceitful communicative acts on the basis of the Cognitive Pragmatics theory (Airenti et al., 1993; Bara, 2010). According to the theory, when two persons communicate, they act based on a pattern of social knowledge shared between the interlocutors. To comprehend the partner’s communicative intentions, a communicative partner must recognize such a stereotyped pattern of shared knowledge. Consider the following example: “Imagine a person enters an office where a woman is waiting and says [1] “It’s icy outside.” While the literal meaning of the sentence is simple to be understood, the woman waiting in the room could be disoriented. She would be able to draw the appropriate inferences and answer appropriately only if she comprehended that [1] meant, for example, to pay attention to the icy ground outside or to close the window, or whatever reason the utterance had been proffered” (Bara, 2010). Within this theoretical framework, it is possible to analyze the inferential processes underlying the comprehension and production of a communicative act uttered with different communicative intentions: sincere, deceitful or ironic. In a sincere communicative act, the speaker expresses knowledge that is consistent with his/her private knowledge. In this case, which is the standard in terms of the inferential process involved, the partner simply has to refer to the interlocutor’s utterance to the knowledge shared with him (see Bara, 2010). In the case of non-standard communications, such as deceitful and ironic communicative acts, more complex inferential processes are involved. Specifically, as previously proposed, “when a speaker utters a deceitful communicative act, her/his intention contrasts with her/his private knowledge, but it does not contrast with the knowledge s/he shares with the partner” (Bosco et al., 2018a). In a deceitful communicative act, the interlocutor “must handle the difference between what is said and what the speaker privately knows” (Bosco et al., 2018a). In the case of irony, the most difficult case to deal with, among those we analyzed, as in the previous case, the interlocutor’s communicative intention is in conflict with her/his private knowledge; however, in this case, it is also in contrast with the piece of knowledge s/he shares with the partner (for a more detailed description see Bara, 2010). According to the theory, these assumptions hold both for communicative acts expressed using both language and extralinguistic means, for example, gestures (see Bara and Tirassa, 1999) and hold both in cases of comprehension and production of a communicative act. A similar trend of increasing difficulty in the comprehension and production of communicative acts proffered with the intention to be sincere, ironic or deceitful has been detected in several studies (Angeleri et al., 2008; Bosco and Bucciarelli, 2008; Gabbatore et al., 2017). However, an alternative explanation for this trend of increasing difficulty could be a primary and increasing role of the EF and ToM involved in the comprehension and production of the abovementioned pragmatic phenomena.

### Cognitive Function and Pragmatic Deficits in Schizophrenia

Few empirical studies in the literature have systematically explored the relationship between ToM and pragmatic ability in schizophrenia; among these studies, Langdon et al. (2002) studied the relationship between ToM and metaphor and irony. The results indicated that individuals with schizophrenia achieved lower scores than controls in both investigated pragmatic tasks. Furthermore, the findings showed that patients’ scores on the ToM items predicted their performance in the comprehension of irony but not metaphors. Mazza et al. (2008) obtained a similar pattern of results. These authors found that patients were impaired in both ToM and irony comprehension, and a correlation was observed between the performances on the two associated tasks. However, Mo et al. (2008) reached different conclusions. These authors used a story comprehension task to investigate the comprehension of utterances proffered with metaphorical and ironical intentions in addition to ToM tasks. In line with previous studies, the findings revealed patient impairments in all tasks investigated. However, in this case, the results did not show a relationship between the performances in the ToM and irony tasks. The patients’ abilities to comprehend metaphors was also found to be associated with EF (Thoma et al., 2009) and proverb comprehension (Sponheim et al., 2003).

Very few studies have investigated the role played by EF and ToM in the pragmatic performance of patients with schizophrenia to disentangle the specific effects of each specific function. Champagne-Lavau and Stip (2010) examined the role of ToM and EF, i.e., shifting, inhibitory control and cognitive flexibility, in patients with schizophrenia in the comprehension of different pragmatic phenomena, including indirect requests and idiomatic and non-idiomatic metaphors. The results indicated that the differences in the performances of the patient and control groups on the pragmatic tasks persisted after ruling out the role of EF. In contrast, after controlling for the role of ToM, the differences persisted for the comprehension of non-idiomatic metaphors but not for the comprehension of indirect speech acts and idiomatic metaphors. In a more recent study, Bambini et al. (2016) investigated the role of cognition, i.e., EF, including WM, planning and processing speed, verbal memory and fluency, IQ, ToM, and pragmatic ability, measured with the Assessment of Pragmatic Abilities and Cognitive Substrates (APACS, Arcara and Bambini, 2016) in a group of clinically stabilized patients with schizophrenia. APACS is a battery that includes assessments of both comprehension and production ability and investigates pragmatic phenomena, such as narrative, ironic and figurative language. The authors found that both cognition and ToM affected patients’ performance in pragmatic comprehension, in contrast to pragmatic production where the regression analysis revealed a significant role only for cognition.

Given the importance of analyzing both the comprehension and production ability and with the aim of extending the investigation to the extralinguistic expressive modality, Parola et al. (2018) analyzed the role played by EF and ToM in sustaining the communicative-pragmatic deficits observed in the pragmatic ability of patients with schizophrenia. The results indicated that after ruling out the role of EF, ToM could predict the patients’ ability in both the comprehension and production aspects of the linguistic tasks but not the extralinguistic tasks, whereas EF did not have any explanatory role in any of the investigated phenomena (linguistic or extralinguistic comprehension and production).

Overall, the state of the evidence concerning the relationships between cognitive functions and ToM and comprehension and production of specific communicative acts in schizophrenia is not completely clear. ToM appears to be the factor more closely related to pragmatic difficulties of patients with schizophrenia. However, the role of ToM has varied greatly across different studies depending on the communicative phenomena investigated (Langdon et al., 2002; Brüne and Bodenstein, 2005; Mazza et al., 2008; Mo et al., 2008; Champagne-Lavau and Stip, 2010; Bambini et al., 2016). In addition, previous studies have investigated the comprehension and production of specific communicative acts by assessing only linguistic expression, while no previous studies have evaluated and compared the recognition and production of different communicative acts in different communicative modalities, i.e., verbal and extralinguistic/non-verbal.

### Present Study

The present study aims to investigate the role of cognitive functions, i.e., general intelligence, selective attention, processing speed, EF and ToM in explaining the ability of patients with schizophrenia to comprehend and produce specific types of pragmatic phenomena, i.e., sincere/literal, deceitful, and ironic communicative acts, expressed through linguistic and non-verbal/extralinguistic means. To the best of our knowledge, this study is the first to investigate the specific role that such cognitive functions play in explaining patients with schizophrenia ability to comprehend and produce communicative acts having different intentions (sincere, deceitful, and ironic) while simultaneously considering both linguistic and extralinguistic/non-verbal expressive means. Consistent with Colle et al. (2013), we hypothesize a decreasing level of patient performance among the investigated tasks in the linguistic and extralinguistic/non-verbal expressive modalities, and both in comprehension and in production, from the simplest to the most difficult: sincere, deceitful, and ironic communicative acts. Finally, we aim to investigate the role played by ToM and EF play in explaining the decreasing trend in the level of communicative performance.

## Materials and Methods

### Participants

A group of 32 individuals with schizophrenia [7 women, 25 men; mean age = 40.17 years, standard deviation (SD) = 10.19; mean education = 10.59 years, *SD* = 2.46] and 32 healthy controls (7 women, 25 men; mean age = 40.28, *SD* = 11.16; mean education = 10.50, *SD* = 2.46) participated in the study. The experimental and control groups were matched by age, education, and gender.

All patients fulfilled the DSM-IV criteria for the diagnosis of schizophrenia (American Psychiatric Association, 2013). The individuals with schizophrenia were chronically ill and clinically stable (no hospitalization during the prior 6 months and no changes in antipsychotic therapy during the prior 3 months). The demographic and clinical characteristics of the participants are presented in Table 1.

**Table 1:** Demographic and clinical data of the patients with schizophrenia and healthy controls.

Variable	Patients		Controls	
Demographic data	Mean	*SD*	Mean	*SD*
Age	40.17	10.77	40.28	11.16
Sex	10.59	2.46	10.50	2.46
Gender (M/F)	25/7		25/7	
**Cut-off test**				
MMSE	27.37	1.68		
AAT	114.81	4.99		
TOKEN	5.91	0.30		
**Clinical measures**				
PANSS total	45.64	19.02		
PANSS positive	18.83	8.89		
PANSS negative	20.28	9.65		


The inclusion criteria for participation in the study were as follows: (1) native Italian speakers; (2) absence of severe cognitive or linguistic deficits as assessed by not exceeding the cut-off scores on the following neuropsychological tests: Mini-Mental State Examination (MMSE; Folstein et al., 1975; cut-off: 24/30), the Token Test, De Renzi and Vignolo, 1962; cut-off: 5/6), and the denomination scale of the Aachener Aphasie test (AAT, Huber et al., 1983; cut-off: no deficit); and (3) attainment of informed consent. The patient symptomatology was assessed using the Positive and Negative Syndrome Scale (PANSS; Kay et al., 1987).

The exclusion criteria for both the experimental and control groups were as follows: (1) evidence of current or prior neurological disorders (e.g., epilepsy); (2) substance or alcohol use disorder; (3) anamnesis of major neurological or neuropsychological disease; (4) hearing or vision problems; and (5) history of head injury.

### Materials

#### Communicative-Pragmatic Assessment

We employed the linguistic and extralinguistic scales of the Assessment Battery for Communication (ABaCo, Angeleri et al., 2012, 2015).

Each scale of the battery evaluates the comprehension and production of different communicative acts: sincere communicative acts (direct and indirect), deceit and irony. In each task, the examiner showed the participants a video-recorded scene in which two semi-professional actors engage in a communicative exchange. In the linguistic scale, the actors in the video communicated using language, and the participants were required to reply verbally to the examiner. In the extralinguistic scale, the actors in the video communicated using performative gestures without any language support, and the participants were required to reply using only communicative gestures. The scale was organized into two subscales, i.e., comprehension and production subscales, which evaluated the comprehension and production of communicative acts, respectively. In the comprehension subscale, after the video was presented, the participants were required to interpret the final statement uttered by one actor in the video. In the production subscale, after the video was presented, the participants were required to produce a communicative act in response to the final statement uttered by one actor in the scene. Each subscale (*linguistic comprehension, linguistic production, extralinguistic comprehension*, and *extralinguistic production*) includes the following experimental tasks: four sincere communicative acts, four deceitful communicative acts, and four ironic communicative acts.

The utterances that were spoken in each scene of the battery have been controlled for the number of words (7 ± 2) to maintain constant memory and attention requirements. The battery was administered individually to each participant and video-recorded, and after administration, it was scored off-line by two independent raters who were blinded to the aims of the study. The psychometric validity of the battery in terms of construct and content validity, as also reliability, has been measured and tested with good results confirming both the validity than the reliability of the tool. For additional details, please see Sacco et al. (2008), Angeleri et al. (2012), and Bosco et al. (2012). Additional details concerning the administration and scoring procedures for the Assessment Battery for Communication are provided in Parola et al. (2016) and Bosco et al. (2017).

Regarding external validity, the battery has been designed such that the items are as ecological as possible in adults. The participants either deals with videorecorded communicative interactions set in everyday contexts or interact with the examiner in short exchanges/conversations. Even if we are unable to provide direct experimental evidence supporting the external validity of the instruments, a recent studies (Bosco et al., 2018a; Parola et al., 2019a) support the convergent validity of a battery with a different instruments measuring functional communication, i.e., the Communication Activities of Daily Living (Holland et al., 1999) and narratives tasks (Marini et al., 2017).

#### Cognitive Functions and Theory of Mind Assessment

The individuals with schizophrenia and healthy controls were administered a battery of cognitive and ToM tasks to assess the cognitive functions most important for the communicative-pragmatic ability. The following cognitive functions were investigated:

Basic cognitive functions:

1. *Attention*: Attentive Matrices (Spinnler, 1987)
2. *General intelligence*: Raven’s Colored Progressive Matrices (RCPM, Raven, 2003)
3. *Processing speed*: Trail Making test Part A (Reitan, 1958)

*Executive functions*:

1. *Working memory*: Disyllabic Word Repetition Test (Spinnler, 1987), Corsi’s Block-Tapping Test (Orsini et al., 1987), and Immediate Recall test (Spinnler, 1987)
2. *Inhibitory control*: Modified Card Sorting test (Nelson, 1976)
3. *Cognitive flexibility:* Trail Making test Part B – Part A (Reitan, 1958)
4. *Planning*: Tower of London (Shallice, 1982)

*Theory of Mind*:

1. *First-order Theory of Mind:* Smarties Task (Perner et al., 1989), Sally & Ann Task (Baron-Cohen et al., 1985)
2. *Advanced Theory of Mind:* a selection of six Strange Stories (Happé,, 1994), excluding stories evaluating communicative aspects.

### Data Analysis

The differences in the pragmatic performances between the individuals with schizophrenia and the healthy controls on the subscales of the ABaCo were examined by submitting the scores obtained on the ABaCo to a 3x2 repeated-measures analysis of variance (ANOVA) with pragmatic phenomena (three levels: sincere, deceitful, and ironic communicative act) as the within-subject factor and group (two levels: patients vs. controls) as the between-subjects factor.

To investigate the significant differences between the cognitive performances of the patients and controls, we performed a series of independent-samples *t*-tests for each cognitive task examined, i.e., general intelligence, selective attention, processing speed, WM, inhibitory control, planning, cognitive flexibility and ToM.

To analyze the role of the cognitive factors and ToM in the pragmatic performance of the individuals with schizophrenia, we performed a hierarchical regression analysis. The relevant predictors were included in the model in three consecutive steps based on their increasing importance in determining pragmatic performance. In particular, in the first stage, we entered basic cognitive factors (general intelligence, selective attention, and processing speed) considered necessary to solve any task. In the second step, EF (WM, cognitive flexibility, inhibitory control and planning) were entered as relevant predictors. In the third and final step, we entered ToM. We conducted regression analyses separately for each of the four subscales of the ABaCo (linguistic comprehension, linguistic production, extralinguistic comprehension, and extralinguistic production).

## Results

The independent-samples *t*-tests showed no significant differences between the experimental and control groups in age (*t*_(62)_ = 0.042, *p* = 0.967) and education (*t*_(62)_ = 0.152, *p* = 0.879).

### Pragmatic Performance

The descriptive statistics of the performance of the patients and controls in the tasks assessing the different pragmatic phenomena, i.e., sincere communicative acts, deceit and irony, evaluated by the ABaCo linguistic and extralinguistic scales are reported in Table 2.

**Table 2:** Mean and standard deviation of the comprehension and production of standard, deceitful, and ironic communicative acts on the linguistic and extralinguistic scales.

ABaCo scales	Communicative acts	Patients (*n* = 32)		Controls (*n* = 32)	
		Mean	*SD*	Mean	*SD*
Linguistic comprehension	Standard	0.85	0.25	0.91	0.18
	Deceit	0.66	0.29	0.90	0.12
	Irony	0.68	0.29	0.86	0.18
Linguistic production	Standard	0.87	0.21	0.95	0.12
	Deceit	0.76	0.23	0.95	0.14
	Irony	0.50	0.30	0.80	0.24
Extralinguistic comprehension	Standard	0.82	0.24	0.91	0.16
	Deceit	0.52	0.33	0.80	0.22
	Irony	0.43	0.31	0.75	0.20
Extralinguistic production	Standard	0.67	0.29	0.95	0.10
	Deceit	0.68	0.28	0.83	0.21
	Irony	0.45	0.36	0.71	0.29


**FIGURE 1:**
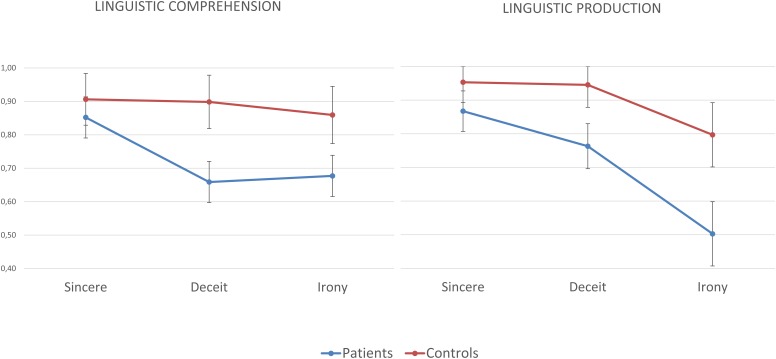
Percentage of correct responses of individuals with schizophrenia and healthy controls in the comprehension and production of sincere, deceitful, and ironic communicative acts on the linguistic scale of the ABaCo.

On the linguistic comprehension subscale, the ANOVA revealed a significant main effect of group (*F*_(1,62)_ = 18.824; *p* < 0.001; *$\eta_{p}^{2}$* = 0.233), indicating that the group of individuals with schizophrenia performed significantly worse than the group of healthy controls on the linguistic comprehension subscale. The main effect of the pragmatic phenomena was also significant (*F*_(2,124)_ = 5.131; *p* = 0.007; $\eta_{p}^{2}$ = 0.076). The linear contrast was significant and revealed a linear decrease in performance depending on the type of communicative act (*F*_(2,62)_ = 7.450; *p* = 0.008; $\eta_{p}^{2}$ = 0.107); the results indicated that the sincere communicative acts were the easiest to understand, followed by deceit and irony (see Table 2). The *post hoc* pairwise comparisons revealed that there were no differences between the patients and controls in the comprehension of the linguistic sincere communicative acts (*p* = 0.32), while significant differences were observed in the comprehension of the deceitful (*p* < 0.001) and ironic communicative acts (*p* = 0.004). See Figure 1.

On the linguistic production subscale, the ANOVA showed a main effect of group (*F*_(1,62)_ = 27.747; *p* < 0.001; $\eta_{p}^{2}$ = 0.309). The group of individuals with schizophrenia performed significantly worse than the group of healthy controls on the linguistic production subscale. The results revealed a main effect of the pragmatic phenomenon (*F*_(2,124)_ = 31.261; *p* < 0.001; $\eta_{p}^{2}$ = 0.335). The results showed a significant linear decrease in performance depending on the type of communicative act considered (*F*_(2,62)_ = 42.818; *p* < 0.001; $\eta_{p}^{2}$ = 0.408); in particular, the production of sincere communicative acts was easier than the production of deceit and irony, which were the most difficult phenomenon to produce (see Table 2). The *post hoc* pairwise comparisons revealed that there were differences between the patients and controls in the production of linguistic sincere (*p* = 0.048), deceitful (*p* < 0.001) and ironic (*p* < 0.001) communicative acts. See Figure 1.

**FIGURE 2:**
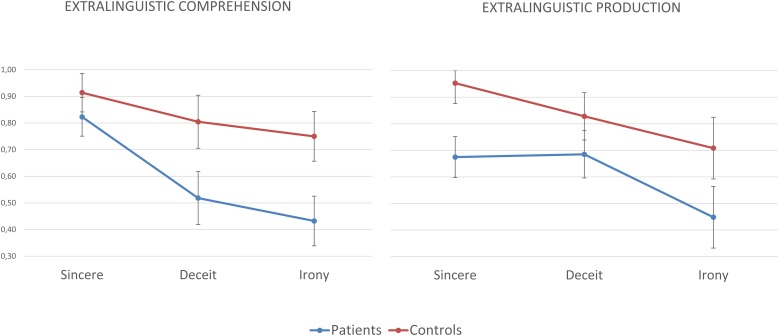
Percentage of correct responses of individuals with schizophrenia and healthy controls in the comprehension and production of sincere, deceitful, and ironic communicative acts on the extralinguistic scale of the ABaCo.

On the extralinguistic comprehension subscale, the ANOVA revealed a main effect of group (*F*_(1,62)_ = 37.739; *p* < 0.001; *$\eta_{p}^{2}$* = 0.378). The individuals with schizophrenia obtained significantly lower scores than the healthy controls on the extralinguistic comprehension subscale. We found a main effect of the pragmatic phenomenon (*F*_(2,124)_ = 21.714; *p* < 0.001; $\eta_{p}^{2}$ = 0.259). The linear contrast showed a linear decrease in scores depending on the pragmatic phenomenon (*F*_(2,62)_ = 37.252; *p* < 0.001; $\eta_{p}^{2}$ = 0.375); this analysis indicated that the comprehension of sincere communicative acts was easier than the comprehension of deceitful and ironic communicative acts, which were the most difficult phenomenon to understand (see Table 2). The *post hoc* pairwise comparisons revealed that there were no differences between the patients and controls in the comprehension of extralinguistic sincere communicative acts (*p* = 0.08), while significant differences were observed in the comprehension of the extralinguistic deceitful (*p* < 0.001) and ironic (*p* < 0.001) communicative acts. See Figure 2.

On the extralinguistic production subscale, the analysis revealed a main effect of group (*F*_(1,62)_ = 22.253; *p* < 0.001; $\eta_{p}^{2}$ = 0.264). Overall, the individuals with schizophrenia had significantly lower scores than the healthy controls on the extralinguistic production subscale. The results revealed a main effect of pragmatic phenomenon (*F*_(2,124)_ = 17.997; *p* < 0.001; $\eta_{p}^{2}$ = 0.225). The linear contrast was significant and showed a linear decrease in performance based on the communicative act considered (*F*_(2,62)_ = 29.763; *p* < 0.001; $\eta_{p}^{2}$ = 0.324); the analysis indicated that the production of sincere communicative acts was easier than the production of deceit and irony (see Table 2). The *post hoc* pairwise comparisons showed that there were differences between the patients and controls in the production of extralinguistic sincere (*p* < 0.001), deceitful (*p* = 0.026), and ironic (*p* = 0.002) communicative acts. See Figure 2.

### Cognitive Functions and Theory of Mind Assessment

The performances of the individuals with schizophrenia and healthy controls in the different cognitive functions and ToM tasks are reported in Table 3. The performance of the individuals with schizophrenia was significantly worse than that of the healthy controls in the following cognitive domains: attention, general intelligence, processing speed, WM, inhibitory control, planning, cognitive flexibility, and ToM (*t-*tests: 2.142 < *t*_(62)_ < 7.17;.0001 < *p* < 0.036).

**Table 3:** Mean and standard deviation of the cognitive and theory of mind tests.

Cognitive functions		Test	Patients		Controls		
			Mean	*SD*	Mean	*SD*	*t*-Value	Level of significance
Basic cognitive ability	Selective attention	*Attentive Matrices*	36.80	8.98	49.07	5.80	–6.49	*p* < 0.001
	Speed processing	*Trail Making test A*	59.86	22.92	32.23	15.53	5.49	*p* < 0.001
	General intelligence	*Raven Matrices*	27.12	6.43	33.53	2.92	–4.09	*p* < 0.001
Executive functions	Working memory	*Verbal Span*	3.52	0.70	4.20	0.90	–3.37	*p* = 0.001
		*Visual Span*	3.85	0.87	5.20	1.14	–5.29	*p* < 0.001
		*Immediate memory*	41.12	22.08	56.22	17.30	–3.05	*p* = 0.003
	Cognitive flexibility	*Trail Making test B-A*	114.36	94.75	32.57	21.41	4.69	*p* < 0.001
	Inhibition	*Nelson*	59.53	35.29	88.60	19.34	–3.70	*p* < 0.001
	Planning	*Tower of London*	21.70	6.04	29.19	3.89	–5.91	*p* < 0.001
Theory of mind	First order ToM	*Smarties*	87.1	34.08	100.0	0.0	–2.14	*p* = 0.036
		*Sally & Ann*	78.13	42.0	100.0	0.0	–2.95	*p* = 0.005
	Second order ToM	*Strange Stories*	65.33	22.46	95.78	8.54	–7.17	*p* < 0.001


### Role of Cognitive Functions and ToM in the Pragmatic Performance of Patients With Schizophrenia and Healthy Controls

The contribution of attention, general intelligence and processing speed—Model 1—in explaining pragmatic performance remains, at best, modest and does not significantly increase the explained variance. The inclusion of Model 2, i.e., EF (inhibitory control, cognitive flexibility, WM, and planning), in the analysis did not significantly increase the level of the explained variance of the comprehension and production of any of the pragmatic phenomena. The inclusion of Model 3, i.e., theory of mind, significantly increased the explained variance only for the linguistic comprehension of deceit (*F*_(1,28)_ = 4.967; *p* = 0.034), for the linguistic production of sincere communicative acts (*F*_(1,28)_ = 8.044; *p* = 0.008), and for the linguistic production of deceit (*F*_(1,28)_ = 4.801; *p* = 0.037) (see Table 4).

**Table 4:** Hierarchical regression analysis of variables predicting the performance of individuals with schizophrenia on the comprehension and production of sincere, deceitful, and ironic communicative acts on both the linguistic and extralinguistic scales: Model 1 (Attention, Speed processing, and General intelligence), Model 2 (WM, Planning, Cognitive flexibility and Inhibitory control), Model 3 (overall Theory of Mind).

DVs	IVs	R^2^	R^2^_Change_	F_Change_	Sig. F_Change_
**Linguistic comprehension**					
Standard	Model 1	0.007	0.007	0.200	0.658
	Model 2	0.040	0.034	1.023	0.320
	Model 3	0.053	0.012	0.362	0.552
Deceit	Model 1	0.007	0.007	0.204	0.655
	Model 2	0.007	0.000	0.003	0.954
	**Model 3**	0.157	0.150	4.967	**0.034**
Irony	Model 1	0.010	0.010	0.315	0.579
	Model 2	0.126	0.115	3.831	0.060
	Model 3	0.132	0.006	0.184	0.671
**Linguistic production**					
Standard	Model 1	0.006	0.006	0.192	0.664
	Model 2	0.076	0.070	2.197	0.149
	**Model 3**	0.282	0.206	8.044	**0.008**
Deceit	Model 1	0.016	0.016	0.484	0.492
	Model 2	0.050	0.034	1.034	0.318
	**Model 3**	0.189	0.139	4.801	**0.037**
Irony	Model 1	0.003	0.003	0.101	0.753
	Model 2	0.005	0.001	0.038	0.847
	Model 3	0.045	0.040	1.171	0.288
**Extralinguistic comprehension**					
Standard	Model 1	0.102	0.102	3.418	0.074
	Model 2	0.130	0.027	0.914	0.347
	Model 3	0.130	0.000	0.009	0.925
Deceit	Model 1	0.000	0.000	0.002	0.964
	Model 2	0.009	0.009	0.257	0.616
	Model 3	0.061	0.052	1.554	0.223
Irony	Model 1	0.008	0.008	0.228	0.636
	Model 2	0.065	0.058	1.796	0.191
	Model 3	0.086	0.020	0.622	0.437
**Extralinguistic production**					
Standard	Model 1	0.036	0.036	1.110	0.300
	Model 2	0.049	0.014	0.413	0.525
	Model 3	0.107	0.057	1.798	0.191
Deceit	Model 1	0.005	0.005	0.137	0.714
	Model 2	0.020	0.015	0.444	0.510
	Model 3	0.080	0.060	1.833	0.187
Irony	Model 1	0.000	0.000	0.006	0.938
	Model 2	0.022	0.022	0.639	0.431
	Model 3	0.022	0.000	0.007	0.934


*The table shows the adjusted regression coefficients (R^2^_Adj_) of each predictor variable, the change in R^2^ after the addition of the planning and theory of mind variables (R^2^_Change_), the change in F (F_Change_) and the significance value (Sig F_Change_). The level of significance for all statistical tests is < 0.005.*

## Discussion

The aim of the present research was to investigate the role that cognitive functions, such as general intelligence, selective attention, processing speed, and especially EF (WM, cognitive flexibility, inhibition, planning) and ToM, have in explaining the ability of individuals with schizophrenia to comprehend and produce communicative acts expressed with different communicative intentions (sincere, deceitful, ironic) and realized through both linguistic and extralinguistic expressive means.

First, we found that in all investigated tasks, the individuals with schizophrenia performed worse than the controls, except for the comprehension of linguistic and extralinguistic sincere communicative acts, which were the easiest tasks analyzed. This pattern of results in line with the relevant literature, confirming that communicative-pragmatic difficulty is a core contributor to the deficits exhibited by most patients with schizophrenia (Langdon et al., 2002; Tavano et al., 2008; Colle et al., 2013; Bambini et al., 2016; Cummings, 2017; Bucca, 2018; Parola et al., 2018; Varga et al., 2018).

More specifically, in line with Colle et al. (2013), our results revealed a decreasing trend in patient performance in managing the different pragmatic tasks investigated, including sincere, deceitful, and ironic communicative acts in both comprehension and production on both the linguistic and extralinguistic scales of the ABaCo (Angeleri et al., 2012). Other studies in the literature have found the same trend of difficulty across different populations, such as patients with left (Gabbatore et al., 2014) and right (Parola et al., 2016) brain damage and traumatic brain injury (Angeleri et al., 2008; Bosco et al., 2018a) and children with typical (Bosco and Bucciarelli, 2008; Bosco et al., 2013) and atypical development, i.e., autism spectrum disorder (Angeleri et al., 2016), and these authors explained the changing difficulty based on the increasing role of the inferential abilities required in the different tasks.

The novelty of the present investigation was to explore the role that different cognitive components have in explaining the ability of patients with schizophrenia to comprehend and produce different types of pragmatic tasks, i.e., sincere, deceitful, and ironic communicative acts. Thus, we examined the performance of patients with schizophrenia and controls in different cognitive domains, i.e., general intelligence, selective attention, processing speed, EF (WM, inhibitory control, planning, and cognitive flexibility) and ToM. In line with the relevant literature (Frith, 1992; Brune, 2005; Harrington et al., 2005; Eisenberg and Berman, 2010; Orellana and Slachevsky, 2013), we found that the participants with schizophrenia had a significantly lower performance than the healthy controls in each task investigated.

Then, we performed a multiple regression analysis to investigate the predictive role of the cognitive factors in explaining the patients’ pragmatic performance on each specific pragmatic phenomenon, i.e., sincere, deceitful, and ironic, on each of the four subscales of the ABaCo (i) linguistic comprehension, (ii) production, (iii) extralinguistic comprehension, and (iv) production. Specifically, we first analyzed the role of basic cognitive factors (general intelligence, selective attention, and processing speed) considered necessary to solve any type of pragmatic tasks. Overall, the role of attention, general intelligence and processing speed in explaining patients’ pragmatic performance was modest and did not appear to significantly explain the patients’ communicative-pragmatic performance. Then, we evaluated the role of EF (WM, cognitive flexibility, planning and inhibitory control) as a relevant predictor. EF can be considered a set of top–down cognitive processes that enable people to control, regulate, and monitor goal-directed behavior and other brain processes necessary to effectively adapt to the environment and achieve goals (Miyake et al., 2000; Diamond, 2013). We entered EF in the second step of the regression analysis because we aimed to evaluate the role of EF in pragmatic performance after controlling for the influence of more basic cognitive functions in the first stage of the model. The percentage of explained variance did not increase significantly with the inclusion of EF, i.e., inhibitory control, cognitive flexibility, WM and planning, in the comprehension and production of any of the pragmatic phenomena investigated. Finally, we evaluated whether ToM significantly explained the pragmatic performance of patients after controlling for the role of EF since some authors have proposed (Bloom and German, 2000) and provided empirical evidence (Pickup, 2008; McDonald et al., 2014; Honan et al., 2015) supporting the claim that most ToM tasks require EF to be correctly solved. ToM was the only factor able to significantly increase the explained variance in the patients’ pragmatic performance but only in the comprehension and production of deceit and production of sincere communicative acts expressed with language. The role of ToM in explaining the ability of patients with schizophrenia in the comprehension of sincere linguistic communicative acts could be explained by the presence of indirect speech acts, a type of communicative act that is considered to require ToM to be understood (Corcoran et al., 1995). It is noteworthy that the comprehension of sincere communicative acts, which is the simplest pragmatic task to solve for the participants with schizophrenia, was explained by ToM ability, while the most complex communicative act, i.e., irony, was not associated with ToM. This result seems to suggest that the role played by ToM in explaining patients’ pragmatic performance is not associated with the inferential complexity of the pragmatic phenomena considered.

The results showing the role of ToM in explaining the ability to deal with deceit is in line with Peskin (1996), who was among the first authors to discuss the importance of this cognitive component in the ability to deal with deceitful speech acts. In contrast, our results did not seem to support previous studies suggesting a causal relationship exists between ToM and the comprehension of verbal irony (Winner and Leekam, 1991). In particular, our results do not support the hypothesis that ToM is the cognitive component that best explains the greater difficulty in comprehending irony compared to that in comprehending deceit (Winner and Leekam, 1991). For similar results and conclusions, see Bosco et al. (2012) and Bosco and Gabbatore (2017a,b). The lack of a ToM causal role in explaining the ability of patients with schizophrenia to process ironic communicative acts also suggests that tasks based on irony comprehension should be used with caution to measure patients’ mentalizing ability (for a deeper discussion, please see Bosco et al., 2018c). ToM is a complex and useful theoretical construct enhancing our understanding of the symptomatology associated with diverse clinical conditions, but ToM appears to be a cognitive domain that does not completely overlap with the pragmatic domain (Laghi et al., 2014; Bambini et al., 2016, Bosco et al., 2018c). For example, as discussed by Bosco et al. (2018c) in greater detail, (pragmatic) inferential ability does not always collapse with the ability to understand others person’s mental states, i.e., ToM. An example is the case of conversational implicatures in which a listener usually infers the speaker’s intended meaning behind the literal one, i.e., what is literally proffered. For example, scalar implicatures rely on quantifiers, such as “some,” “all,” etc. For example, the interpretation of “On Sofia’s bed, some of the teddy bears are red” appears to imply that not all teddy bears are red. In cases as the one mentioned above, no assumptions about beliefs or other types of mental states appear to be required, thus making the ability to comprehend implicatures based only on inferential processes.

Notably, such processes might also involve ToM abilities. This issue deserves further empirical investigation in order to be clarified.

In summary, the novelty of the present study is the investigation of the specific role played by specific cognitive functions in explaining the ability of patients with schizophrenia to comprehend and produce communicative acts with different underlying intentions (sincere, deceitful, and ironic) while simultaneously considering both linguistic and extralinguistic/non-verbal expressive means. Our most original result is that the linear increasing trend of difficulty detected in the comprehension and production of sincere, deceitful, and ironic communicative acts expressed through both language and extralinguistic (non-verbal) expressive means seems to not be explained by the increasing role of a specific set of cognitive components (i.e., basic cognitive abilities + EF + ToM). In line with previous investigations (Angeleri et al., 2008; Colle et al., 2013), we suggest that the increasing trend of difficulty among the tasks investigated, i.e., sincere, deceitful, and ironic communicative acts, could be better explained by the increasing inferential ability necessary to comprehend and produce each task. In particular, our findings appear to not be in favor of the role of ToM in explaining the increasing difficulty of patients with schizophrenia in recognizing ironic communicative acts compared to deceitful ones. Indeed, we found that ToM was associated with the patients’ difficulty in producing and comprehending deceit, but ToM was unable to explain the patients’ increasing difficulty in managing irony compared to deceit. Thus, we hypothesize that the inferential chain specifically explains the increasing trend of difficulty detected, but further empirical research is necessary to support this hypothesis. For example, future studies could use specific tasks to provide a direct measure of (pragmatic) inferential ability (e.g., scalar implicature task) and then evaluate the presence of covariance patterns between such a measure and different types of pragmatic tasks, such as irony comprehension.

Our results seem to support a recent study showing that impairments in pragmatics is a core and specific deficit of patients with schizophrenia (Bambini et al., 2016). Specifically, the authors administered the APACS test (Arcara and Bambini, 2016) in addition to a set of cognitive and ToM tasks and measured verbal memory, WM, verbal fluency, processing speed, and EF (planning) and found that the pragmatic deficits do not overlap with other cognitive deficits in more than 30% of the patients investigated. These results indicate that pragmatic impairment should not be merely reduced to the underlying cognitive deficits and highlight the domain specificity of pragmatic ability. This type of empirical evidence is particularly important given Pawełczyk et al. (2018) proposal. The authors investigated the presence of pragmatic impairment in patients with schizophrenia and their healthy first-degree relatives and argued that pragmatic dysfunction could be considered a vulnerability marker in patients with schizophrenia and that its assessment could help diagnosis during the early stage of the illness.

In summary, the results of our study suggest that in addition to the domain usually considered in the clinical assessment of the cognitive impairment of patients with schizophrenia, the role of pragmatic and specifically the role of the (increasing) inferential processes involved in a specific pragmatic phenomenon should be considered to better comprehend the communicative difficulty that characterizes such pathology.

Finally, despite the limitations, the present study has relevant implications for the assessment of cognitive abilities in patients with schizophrenia, highlighting the importance of considering patients’ pragmatic symptoms as a distinct domain of assessment with respect to other cognitive domains, such as EF and ToM. This study may also provide useful suggestions for the development of rehabilitative strategies aiming to help patients recover and enhance their communicative performance since recent studies have revealed a correlation between pragmatic ability and communicative effectiveness in daily life (Bosco et al., 2018b) and the perception of the quality of life (Bambini et al., 2016). Indeed, in addition to other relevant interventions, the ability to manage inferences should be considered in planning therapeutic rehabilitative programs (see, for example, Gabbatore et al., 2015; Bosco et al., 2016).

## Ethics Statement

This study was carried out in accordance with the recommendations of ‘A.S.L. To2 ethics committee’ with written informed consent from all subjects. All subjects gave written informed consent in accordance with the Declaration of Helsinki. The protocol was approved by the ‘A.S.L. To2 ethics committee.’

## Author Contributions

FB experimental design and paper writing. LB contact with patients. AP statistical analyses and administration of experimental material.

## Conflict of Interest Statement

The authors declare that the research was conducted in the absence of any commercial or financial relationships that could be construed as a potential conflict of interest. The reviewer BB declared a shared affiliation, with no collaboration, with several of the authors, FB and AP, to the handling Editor at the time of review.
